# Evidence of indirect symbiont conferred protection against the predatory lady beetle *Harmonia axyridis* in the pea aphid

**DOI:** 10.1186/s12898-017-0136-x

**Published:** 2017-07-11

**Authors:** Jennifer L. Kovacs, Candice Wolf, Dené Voisin, Seth Wolf

**Affiliations:** 1 Spelman College, 350 Spelman Lane, S.W., Atlanta, GA 30314 USA; 2 University of Vermont, Larner College of Medicine, 89 Beaumont Ave, Burlington, VT 05405 USA; 3 Neuroscience Institute, Georgia State University, Petit Science Center, Atlanta, GA 30303 USA

**Keywords:** Protective symbiosis, Facultative symbionts, Indirect fitness effects, Predation

## Abstract

**Background:**

Defensive symbionts can provide significant fitness advantages to their hosts. Facultative symbionts can protect several species of aphid from fungal pathogens, heat shock, and parasitism by parasitoid wasps. Previous work found that two of these facultative symbionts can also indirectly protect pea aphids from predation by the lady beetle *Hippocampus convergens.* When aphids reproduce asexually, there is extremely high relatedness among aphid clone-mates and often very limited dispersal. Under these conditions, symbionts may indirectly protect aphid clone-mates from predation by negatively affecting the survival of a predator after the consumption of aphids harboring the same vertically transmitted facultative symbionts. In this study, we wanted to determine whether this indirect protection extended to another lady beetle species, *Harmonia axyridis*.

**Results:**

We fed *Ha. axyridis* larvae aphids from one of four aphid sub-clonal symbiont lines which all originated from the same naturally symbiont free clonal aphid lineage. Three of the sub-clonal lines harbor different facultative symbionts that were introduced to the lines via microinjection. Therefore these sub-clonal lineages vary primarily in their symbiont composition, not their genetic background. We found that aphid facultative symbionts affected larval survival as well as pupal survival in their predator *Ha. axyridis*. Additionally, *Ha. axyridis* larvae fed aphids with the *Regiella* symbiont had significantly longer larval developmental times than beetle larvae fed other aphids, and females fed aphids with the *Regiella* symbiont as larvae weighed less as adults. These fitness effects were different from those previously found in another aphid predator *Hi. convergens* suggesting that the fitness effects may not be the same in different aphid predators.

**Conclusions:**

Overall, our findings suggest that some aphid symbionts may indirectly benefit their clonal aphid hosts by negatively impacting the development and survival of a lady beetle aphid predator *Ha. axyridis*. By directly affecting the survival of predatory lady beetles, aphid facultative symbionts may increase the survival of their clone-mates that are clustered nearby and have significant impacts across multiple trophic levels. We have now found evidence for multiple aphid facultative symbionts negatively impacting the survival of a second species of aphid predatory lady beetle. These same symbionts also protect their hosts from parasitism and fungal infections, though these fitness effects seem to depend on the aphid species, predator or parasitoid species, and symbiont type. This work further demonstrates that beneficial mutualisms depend upon complex interactions between a variety of players and should be studied in multiple ecologically relevant contexts.

**Electronic supplementary material:**

The online version of this article (doi:10.1186/s12898-017-0136-x) contains supplementary material, which is available to authorized users.

## Background

Symbionts, both obligate and facultative, can affect the fitness of their hosts in a variety of ways. Symbionts can have little or no effect on their host (often called commensals). While, others can negatively affect the fitness of their hosts, and still other symbionts are beneficial and can provide diverse fitness advantages to their hosts [1]. For example, in *Drosophila neotestacea*, a vertically transmitted symbiotic *Spiroplasma* bacteria protects its female fly hosts from being sterilized by a parasitic *Howardula* nematode [2]. While in herbivorous *Megacopta* stinkbugs, symbiotic bacteria seem to determine what host plants their hosts can utilize. Normally *Megacopta cribaria* suffers high mortality when reared on legumes. However when they are provided with symbiont capsules from the legume pest *Megacopta punctatissima* and obtain its symbiotic gut bacteria, *M. cribaria* are then able to utilize legumes as host plants [3]. In both of these examples, as well as in other cases, symbionts can have impacts beyond just their host species.

Symbionts can have effects across trophic levels, food webs, and ecological communities [2, 4–14]. They can mediate inter-specific interactions, such as competition, parasitism, and predation. For example, rove beetles in the genus *Paederus* harbor *Pseudomonas* symbionts which produce the toxic amide pederin. Pederin is highly cytotoxic and blocks protein synthesis inhibiting mitosis [15, 16]. In humans, pederin can result in itching lesions and dermatitis when it comes in contact with skin, often through the crushing of beetles [17]. *Paederus* females use their eggshells to vertically transfer *Pseudomonas* to their offspring. While pederin-provisioned larvae are just as likely to be attacked and eaten by predatory insects as *Paederus* larvae without pederin, the pederin does provide protection from predation by spiders [18]. Wolf spiders avoid *Paederus* larvae and eggs with pederin as well as *Drosophila* flies artificially provisioned with the pederin toxin [18]. In this example, the *Pseudomonas* symbiont provides a very direct defensive benefit to its host against predation by wolf spiders.

In aphids, facultative symbionts are not essential to aphid growth or survival and are not present in all aphid populations, but these facultative symbiotic bacteria can provide protection against pathogens, heat stress, and parasitoids [1, 11, 13, 19–25]. For example, when parasitoid wasps lay their eggs in aphids harboring the *Hamiltonella defensa* symbiont, those eggs fail to develop or the larvae experience very high mortality rates [26]. Additionally, Rothacher et al. [13] observed shifts in the composition of parasitoid wasp species within a natural community when black bean aphid *Aphis fabae* harbored the protective symbiont *H. defensa*. This demonstrates the potential that symbionts, particularly defensive symbionts, can have on the structure and the diversity of food webs.

A recent study found that the survival of predatory lady beetle *Hippodamia convergens* larvae was decreased when they were fed pea aphids with facultative symbionts *H. defensa* and *Serratia symbiotica* [14]. Rather than directly protecting aphids from immediate predation, as in the case of the rove beetles, aphid symbionts may be providing indirect protection to their aphid hosts. Aphids live in tightly clustered, extremely highly related clonal groups during the parthogenetic portion of their annual life cycle [27]. By lowering predator survival, aphid symbionts could also lower the overall risk of predation for a group of clonal aphids, thereby providing indirect protection against predation by lady beetles [28–30]. We were interested in determining whether three aphid symbionts (*H. defensa, S. symbiotica,* and *Regiella insecticola*) negatively affected the survival of a second species of predatory lady beetle, *Harmonia axyridis*. Based on previous findings in *Hi. convergens,* we predicted that aphid secondary symbionts would lower predator survival and negatively impact larval weight and development time [14]. Our current findings suggest that multiple aphid symbionts also provide indirect protection from a second species of predatory lady beetles.

## Methods

### Survival experiments

Adult lady beetles (*Ha. axyridis*) were collected at Spelman College in Atlanta, GA, USA. Lady beetles were kept in mixed sex groups and maintained at 25 °C with a light regime of 16:8 Light:Dark. Adult beetles were fed aphids from genetically identical asexual aphid lineages harboring either no facultative symbionts (aphid line 5AO), the facultative symbiont *S. symbiotica* (5AR), *H. defensa* (5AT), or *R. insecticola* (5AU). All four aphid symbiont lineages (5A0, 5AR, 5AU, and 5AT) were established from the same naturally uninfected 5A clone (collected in Madison, WI, USA, June 1999). Facultative symbionts were introduced to the 5A clone through microinjection of body fluids containing symbionts (5AR & 5AU [31], 5AT, [10]). Prior to starting the experiment, lines were screened for the respective facultative symbionts using qPCR. In addition, *H. defensa* is associated with a phage, APSE, that is known to have important effect on some *Hamiltonella*-conferred phenotype. We used PCR to confirm the presence of APSE in our *Hamiltonella*-infected line ([32] unpublished data). Aphids were reared on fava seedlings (*Vicia faba* L.) at 20 °C with a light regime of 18:6 Light:Dark. New aphid bearing plants were supplied to the adult lady beetles daily.

After a week of captivity, we began removing lady beetle egg clutches from the adult cage daily and placing egg clutches in Petri dishes. Once eggs hatched, larvae were separated into individual Petri dishes. Larvae were fed aphids from the same aphid line containing the same facultative symbiont that their parents ate. We note that due to this experimental design we are unable to determine whether our observed results are due to the feeding of the symbiont to the mother (maternal effects) or to the larvae. In either case, any observed survival effect would be due to the symbiont type which was the same in both the maternal and the larval diet. Larvae were raised individually to prevent cannibalism and competition between individuals. Larvae were fed fresh aphids ad libitum, and moist cotton balls were supplied and replaced as needed. All larvae were provided approximately the same number of third and fourth instar aphids every day (~10–15 aphids per feeding during instars 1 and 2 and ~15–20 aphids during instars 3 and 4), and no major differences in feeding rates were observed between the four experimental groups, though precise daily feeding rates were not recorded in this study. Preliminary work determined that under our lab conditions *Ha. axyridis* larval development lasted 19 days from hatching to pupation. Instars one and two took 7 days, the third instar 5 days, and the fourth instar 8 days. However, exact dates for each instar molt for each individual were not recorded in this study. Time to pupation, time to emergence from pupation, and time to death were recorded daily. Larvae were weighed at day 8, and adult lady beetles were weighed upon emergence prior to additional feeding. After adult emergence individuals were sexed using dimorphic features of the distal margin of the final abdominal sternite. In a single trial lasting 40 days, 305 lady beetle larvae were observed from hatching to either adult emergence or death. Seventy-five lady beetle larvae were fed 5AT aphids, 76 were fed 5AR aphids, 75 were fed 5AU and 79 were fed 5AO aphids. All data generated and analyzed in this study are available in supplementary material files associated with this publication (Additional file 1: Data S1).

### Statistical analyses

To determine whether the type of aphid symbiont in the larval diet affected overall lady beetle survival from hatching to adult emergence, we used a generalized linear model with a logit linked binomial distribution (reached adult stage = 1, died prior to reaching adult stage = 0) with “symbiont”, “larval weight at day 8”, and the interaction between the two as factors. The interaction term was included in the final model based on the resulting delta AICc (Akaike information criterion adjusted for sample size) scores. The model with the lowest AICc score was considered to be the “best” model. Non-significant interaction terms were retained when the delta AICc between the full model (run with the interaction term and all other factors) and the “best” model was less than 2 and removed when it was more than 2. Post-hoc pairwise contrast analyses were performed to determine whether observed differences in pupal survival between aphid sub-clonal lines were significant.

We then further broke down survival, looking first at survival to pupation and then survival from pupation to adult emergence. The effects of aphid symbionts in the diet of *Ha. axyridis* larvae on larval survival from hatching to pupation were analyzed using a right censored Cox’s proportional hazard model with “symbiont”, “larval weight at day 8”, and their interaction term as factors after testing the assumption of proportional hazards using cox.zph of the survival package in R [33]. All three factors were retained in the final model based on the delta AICc score between the two models (Δ AICc <2). In the case of ties, Breslow likelihood was used. In addition to the survival analysis, several correlations were run to determine the biological relevance of larval weight at day 8. Specifically, we tested for correlations between (1) larval weight at day 8 and the day an individual died as a larvae and (2) larval weight at day 8 and pupation day for those that successfully pupated. We ran an ANOVA and Tukey–Kramer HSD tests to determine whether aphid symbiont significantly affected larval weight at day 8.

Survival from pupation to adult emergence (eclosion) was analyzed using a generalized linear model with a logit linked binomial distribution run on a subset of the 232 individuals that successfully pupated. Once individuals reach pupation, it is difficult to determine whether they are alive or not. Therefore pupa were determined to have died when they did not emerge as adults after 2 weeks; pupa were recorded as either successfully emerging as adults (=1) or as being dead (=0). Based on the delta AICc scores, “symbiont” and “larval weight at day 8” were included as factors in this analysis (Δ AICc >2). Post-hoc pairwise contrast analyses were performed to determine whether observed differences in pupal survival between aphid sub-clonal lines were significant.

For those individuals surviving to pupation and to adulthood, general linear models (standardized least square) were used to determine the effect of “symbiont” and “larval weight at day 8” on lady beetle development time, both from hatching to pupation and from pupation to adult emergence separately. The interaction term between the two factors was removed from both final models based on the Δ AICc score between the two models being greater than 2. We used ANOVA’s and post hoc Tukey–Kramer HSD tests to determine which groups were significantly different from one another. Correlations were also done to assess whether there were significant correlations between larval weight and development times.

For analyses of adult weight, male and female beetles were analyzed separately. Adult weights at emergence were standardized to have a mean of zero and a standard deviation of one for males and females separately. To determine the effect of aphid facultative symbionts on male adult weight, general linear models (standard least square) were run with “symbiont”, “larval weight at day 8”, and the interaction term as factors. For female adult weight the same models were used, but the interaction term was removed from the final model based on the Δ AICc score between the two models being greater than 2. We used ANOVA and post hoc Tukey–Kramer HSD tests to determine which groups were significantly different from one another. Correlations were also done to assess whether there were significant relationships between larval weight and adult weight in both sexes. Additionally, we tested whether the sex ratio of emerging adults was significantly different from the expected 0.50 probability for each sex using a two-sided Chi square test. All statistical analyses were performed in JMP^®^ Pro 13.0.

## Results

**Fig. 1:**
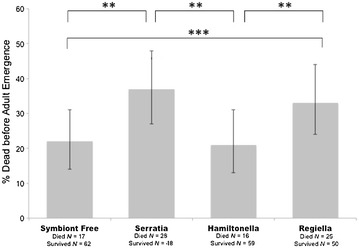
Survival from hatching to adult emergence depending on larval diet of *Harmonia axyridis.* Percentages of survival and 95% confidence intervals are shown. * indicates significant differences at a 0.05 < *p* < 0.01 level, ** at a 0.09 < *p* < 0.00011, ***p < 0.0001 obtained using post hoc pairwise contrasts

We found a significant effect of aphid symbiont in the diet of *Ha. axyridis* larvae on survival from hatching to adult emergence. Both aphid symbionts present in the larval diet of these beetles and their weight at day 8 significantly affected overall survival to adult emergence (Fig. 1; Table 1). In the post hoc pairwise contrast analyses, we found that individuals that were fed aphids harboring the *Serratia* symbiont and the *Regiella* symbiont were more likely to die prior to reaching adult emergence than those fed aphids with the *Hamiltonella* symbiont or those fed aphids without symbionts (*Serratia/Hamiltonella*: *χ* ^2^ = 9.21, *p* = 0.002; *Serratia/*symbiont free: *χ* ^2^ = 9.85, *p* = 0.002; *Regiella/Hamiltonella*: *χ* ^2^ = 8.14, *p* = 0.004; *Regiella/*Symbiont free: *χ* ^2^ = 11.03, *p* < 0.0001). There was no significant difference in survival between larvae fed aphids with *Hamiltonella* and those without symbionts (*χ* ^2^ = 0.007, *p* = 0.93), nor was there a difference in survival rates between those fed aphids with *Regiella* and *Serratia* (*χ* ^2^ = 0.005, *p* = 0.95).

**Table 1:** Summary of results of statistical models

Dependent variable	Name of statistical test	Statistic	Whole model	Symbiont	Weight at larval day 8	Symbiont × weight interaction
Overall survival from hatching to adult emergence	Generalized linear model (binomial distribution)	Likelihood ratio *χ* ^2^	28.10**	24.39***	5.50*	7.62
Larval survival	Cox’s proportional hazards model	Likelihood ratio *χ* ^2^	13.36	8.80*	1.16	5.55
Pupal survival	Generalized linear model (binomial distribution)	Likelihood ratio *χ* ^2^	7.68	7.67*	0.11	0.50^a^
Larval development time	General linear model (least square)	F_4,221_	26.85***	12.32***	67.60***	0.99^a^
Pupal development time	General linear model (least square)	F_4,212_	1.70	1.20	2.81	1.18^a^
Female adult weight at emergence	General linear model (least square)	F_4,77_	3.81**	4.95**	0.12	3.76^a^
Male adult weight at emergence	General linear model (least square)	F_7,87_	4.35**	2.15	0.20	7.57***

Significant results for statistical analyses are indicated by asterisks

* 0.05 < *p* < 0.01, ** 0.009 < *p* < 0.00011, *** *p* < 0.0001

^a^Based on resulting AICc scores, non-significant interaction terms were removed from the final model when the Δ AICc >2. The results of the final model are reported for the other terms

**Fig. 2:**
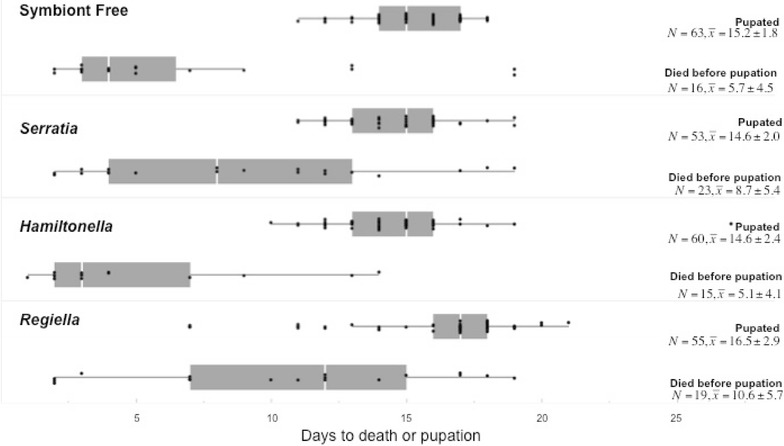
Larval survival depending on larval diet of *Harmonia axyridis.* Larval survival was measured from hatching day to larval death. In the *box* and *whisker plots*, the *bottom* and the *top* of the *box* denote the first and third quartile. The band inside the *box* denotes the second quartile (median) and the *whiskers* denote the maximum and minimum of the data. Points outside of the *whiskers* are outliers. All sample sizes (*N*) are noted for those the pupated or died prior to pupation and means and standard deviations are provided

Feeding *Ha. axyridis* larvae a diet of aphids with the facultative symbionts *Serratia* (5AR) and *Regiella* (5AU) significantly lowered larval survival to pupation when compared to those larvae fed symbiont-free aphids (Fig. 2; Table 1). Larvae fed aphids with *Serratia* were 9.32 times more likely to die before reaching pupation than those fed symbiont free aphids (*p* = 0.013), and larvae fed aphids with *Regiella* were 7.47 times more likely to die as larvae than larvae fed aphids without facultative symbionts (*p* = 0.022). While not statistically significant, larvae fed *Serratia* were 4.46 times more likely to die as larvae as those fed aphids with *Hamiltonella* (*p* = 0.06), and larvae fed *Regiella* were 3.57 times more likely to die as larvae as those fed aphids with *Hamiltonella* (*p* = 0.10). Larval weight at day 8 was included as a factor in the Cox’s proportional hazard model due to the results of our correlations, as well as the difference in the AICc scores between the two models (Δ AICc >2). While larval weight at day 8 was not correlated with the day an individual died as a larvae (*r* ^*2*^ = 0.03, *p* = 0.33), it was significantly negatively correlated with pupation day for those individuals surviving to pupation (*r* ^*2*^ = 0.22, *p* < 0.0001). Additionally, larvae fed aphids with the *Serratia* symbiont weighed significantly more than those fed other aphids (*F*_3,256_ = 9.54, *p* < 0.0001, Tukey–Kramer HSD, *p* < 0.0002 for all comparisons to *Serratia*). However, when “symbiont” and larval weight were both added to the model, larval weight at developmental day 8 did not appear to have a significant effect on larval survival, nor was there a significant interaction between the type of aphid eaten and larval weight, suggesting that aphid symbiont is largely responsible for the differences in larval survival measured in this experiment.

**Fig. 3:**
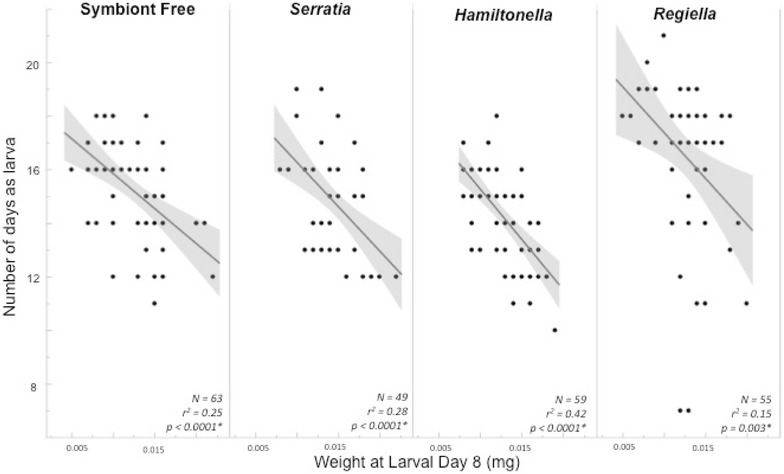
Larval development time depending on larval diet of *Harmonia axyridis. Lines* represent best fit for each group of *Ha. axyridis* larvae fed a different aphid sub-clonal line either without symbionts or with one of the three tested symbiont types. The *shaded areas* are 95% confidence curves

For those individuals surviving to pupation, both the type of symbiont present in the larval diet (“symbiont”) and larval weight significantly affected time spent as a larvae prior to pupation (Fig. 3; Table 1). Overall, there was a negative correlation between larval weight and the number of days spent as a larva prior to pupation, meaning individuals that weighed less at larval day 8 spent more time as larvae prior to pupation than those that were heavier at larval day 8 (*r* ^*2*^ = 0.216, *p* < 0.0001). Additionally, larvae fed aphids harboring *Regiella* (5AU) spent significantly longer as larvae than the other three experimental groups (*F*_3,223_ = 11.26, *p* < 0.0001, Tukey–Kramer HSD, *Regiella/Hamiltonella p* < 0.0001, *Regiella/Serratia p* < 0.0001, *Regiella*/symbiont free *p* = 0.004).

**Fig. 4:**
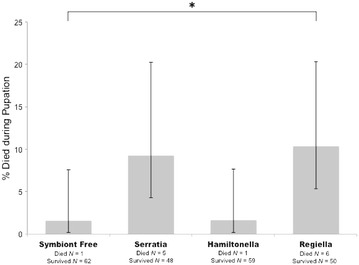
Survival from pupation to adult emergence depending on larval diet of *Harmonia axyridis.* Percentages of survival and 95% confidence intervals are shown. * indicates significant differences at a 0.05 < *p* < 0.01 level obtained using post hoc pairwise contrasts

Individual survival from pupation to the adult stage was also affected by larval diet, though we note that overall pupal mortality was low ranging from 2 to 11% (Fig. 4). Larval weight did not significantly effect pupal survival, nor was there a significant interaction between the two effects (Fig. 4; Table 1). Post-hoc pairwise contrast analyses revealed that pupae that as larvae were fed aphids with *Regiella* were significantly more likely to survive to adult emergence than those fed aphids without symbionts (*Regiella/*symbiont free: *χ* ^2^ = 4.82, *p* = 0.03). All other comparisons were not significantly different from one another (*p* > 0.05).

**Fig. 5:**
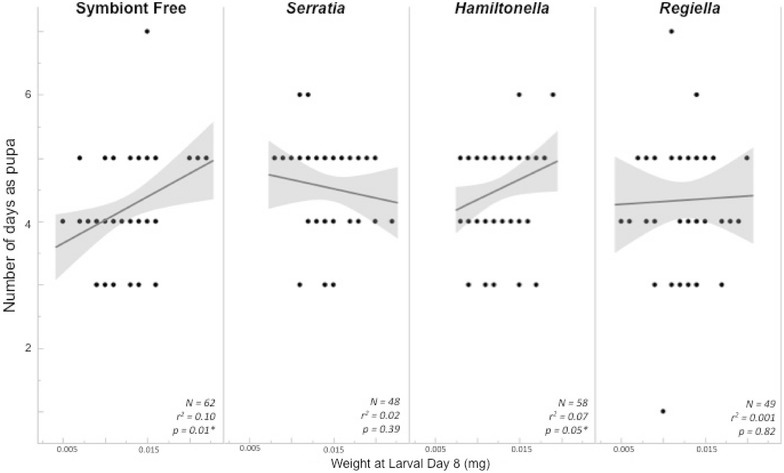
Pupal development time depending on larval diet of *Harmonia axyridis. Lines* represent best fit for each group of *Ha. axyridis* larvae fed a different aphid sub-clonal line either without symbionts or with one of the three tested symbiont types. Number of days spent as a pupa is shown on the *y-axis*. The *shaded areas* are 95% confidence curves

None of the measured variables affected the number of days an individual spent as a pupa prior to emergence as an adult, though the length of pupal development and larval weight at day 8 were significantly correlated for two of the symbiont groups (Fig. 5; Table 1). We saw no correlation between larval weight and the number of days spent as a pupa (*r* ^*2*^ = 0.006, *p* = 0.24). We also saw no effect of aphid symbiont type eaten on time to adult emergence (*F*_3,213_ = 0.843, *p* = 0.47). However, for individuals fed aphids without symbionts and those fed aphids with the *Hamiltonella* symbiont there were significant correlations between larval weight at day 8 and the length of time spent as a pupa prior to adult emergence (Fig. 5; symbiont free: *r* ^*2*^ = 0.10, *p* = 0.01; *Hamiltonella*: *r* ^*2*^ = 0.07, *p* = 0.05). There were no correlations between larval weight and pupal development length for individuals fed aphids with the *Serratia* or *Regiella* symbionts (Fig. 5; *Serratia: r* ^*2*^ = 0.02, *p* = 0.39, *Regiella: r* ^*2*^ = 0.001, *p* = 0.82).

**Fig. 6:**
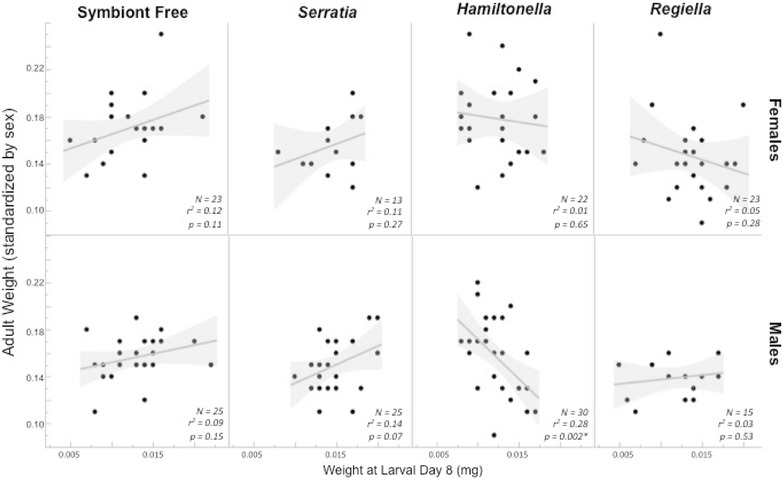
Female and male adult weight at emergence depending on larval diet of *Harmonia axyridis. Lines* represent best fit for each group of *Ha. axyridis* larvae fed a different aphid sub-clonal line either without symbionts or with one of the three tested symbiont types by sex. The *shaded areas* are 95% confidence curves

Aphid symbionts in the lady beetle larval diet (“symbiont”) also affected weight at adult emergence for female lady beetles, but not males. Overall, larval weight was not correlated with adult weight at emergence in either sex (Fig. 6; Table 1). Females fed aphids with *Regiella* symbionts (5AU) weighed significantly less as adults than those fed aphids without symbionts or with *Hamiltonella* (*F*_3,78_ = 5,14, *p* = 0.003, Tukey–Kramer HSD, *Regiella*/*Hamiltonella*: *p* = 0.003, *Regiella*/symbiont free: *p* = 0.036). Additionally, in males, there was a significant interaction between larval weight and aphid symbiont on adult weight. This appears to be due to a negative correlation between larval weight and adult weight for male lady beetle larvae fed aphids harboring the *Hamiltonella* symbiont (5AT), while for the three other male lady beetle experimental groups there was no correlation, negative or positive, between larval weight and adult weight (Fig. 6; *Hamiltonella*: *r* ^*2*^ = 0.288, *p* = 0.002, *Regiella: r* ^*2*^ = 0.031, *p* = 0.532, *Serratia: r* ^*2*^ = 0.135, *p* = 0.071, no symbiont*: r* ^*2*^ = 0.087, *p* = 0.153).

There was no evidence that larval aphid diet affected the survival of males and females differently. The resulting sex ratio of surviving adults was not significantly different from the expected 50/50 sex ratio for any of the experimental groups ([34], two-sided *χ* ^2^, *p* > 0.05 for all tests).

## Discussion

Two of the three aphid symbionts used in this study significantly decreased both the larval and pupal survival of the aphid predator *Ha. axyridis*. Specifically, when larvae were fed aphids harboring either the *Serratia* or *Regiella* symbiont, they were significantly less likely to survive as larvae or to emerge from pupation as adults than those larvae that were fed aphids without symbionts or with the *Hamiltonella* symbiont. Additionally, *Ha. axyridis* larvae fed aphids with the *Regiella* symbiont had significantly longer larval development times than those fed aphids with no symbionts or the *Hamiltonella* or *Serratia* symbionts. Finally, adult females that had been fed *Regiella*-harboring aphids as larvae weighed significantly less than other adult female lady beetles.

These results are particularly striking due to the clonal nature of these asexual aphid lineages. All four symbiont types were established from the same clonal line (5A) and have been maintained under environmental lab conditions that ensure parthenogenetic reproduction since collection. While some mutations may have accumulated in the four sub-clonal lines since the injection of symbionts [35], the major difference between these sub-clonal aphid lines is the presence and type of symbiont. This strongly suggests that the presence of the *Regiella, Serratia*, and *Hamiltonella* aphid symbiont in the larval diet of *Ha. axyridis* are affecting lady beetle larval and pupal survival, rather than another factor associated with the aphid, like genotype.

Aphid facultative symbionts have been found to benefit their aphid hosts in a variety of ways, including protecting them from heat shock and from parasitism [1]. In nearly all of these cases, the protection directly benefits the individual aphid harboring the symbiont. In this experiment, aphid symbionts affect the fitness of the aphid predator, but only after the individual harboring the symbiont has been eaten. In this scenario, the aphid symbionts are not providing direct protection to their hosts, but rather may be providing indirect protection to their clonal siblings harboring the same vertically transmitted facultative symbionts that are clustered nearby. Aphids reproduce parthenogenetically for the majority of their annual life cycle and in the summer months live in patches of genetically identical (or nearly identical) individuals [36]. Female lady beetles lay their eggs near aphid patches, and there is evidence to suggest that lady beetles do not disperse far during their larval and pupal stages [37]. With low dispersal of both the predator and the prey and high relatedness among clumps of aphids, we suggest that while the aphids that are eaten by lady beetles do not themselves benefit from their symbionts, by reducing the survival of local predators other aphids in their patch, clone-mates may receive indirect fitness benefits from their aphid symbionts [28, 38].

Other behaviors that may have indirect fitness effects have been observed among clusters of aphid clone-mates, and particular interest has been paid to the role of alarm pheromone in altruistic aphid defense behaviors [28, 39]. Droplets of aphid alarm pheromone that aphids smear on the predator only after being physically attacked, while not directly benefiting the individual emitting the alarm signal, may increase the likelihood that the highly related clone-mates around them escape predation [39]. Grain aphids that secrete alarm pheromones when being parasitized by wasps do not themselves seem to benefit from this action; it doesn’t reduce parasitism. However, once smeared with the alarm pheromone, parasitoid wasps spend significantly more time grooming and less time ovipositing. The foraging efficiency of the parasitoid is also greatly reduced due to the defensive behaviors other aphids exhibit when exposed to the previously released alarm pheromone, such as dropping from the plant or kicking their legs [28]. All together, this can reduce parasitism within patches of clone-mates thereby providing indirect fitness benefits to the parasitized aphid. In all of these cases, lowering the predator/parasite foraging efficiency and efficacy results in a lower overall risk of predation/parasitism for a population of genetically identical aphids.

In a previous study, we found both the *Serratia* and *Hamiltonella* aphid symbionts negatively impacted the larval survival of another predatory lady beetle species, *Hi. convergens* [14]. Additionally, female *Hi. convergens* larvae fed aphids with *Serratia* or *Hamiltonella* symbionts weighed more as adults. While these experiments were not run simultaneously making direct comparisons difficult, our current findings do suggest a pattern in which the presence of aphid symbionts can significantly decrease the survival of aphid predators. It also appears that each symbiont may affect each predator differently. For example, female adult weight was significantly higher in *Hi. convergens* fed aphids with *Serratia* as larvae, but there was no difference in *Ha. axyridis* female adult weight between those fed aphids with *Serratia* and those fed symbiont free aphids. Additionally, while *Hamiltonella* negatively impacted the larval survival of *Hi. convergens*, there was no negative effect of the *Hamiltonella* symbiont on the survival of *Ha. axyridis*. Though these differences in the effects of several aphid symbionts in these two predatory lady beetle species may be due to other unmeasured differences in these two separate sets of experiments, they may also suggest variation in the mechanisms by which symbionts affect different aphid predators.

Other studies have found differences in aphid symbiont-conferred resistance to depend on a number of factors, including aphid host species, parasitoid species, other symbionts which may be present in the host, and bacteriophages associated with some symbionts [1, 10, 11, 19–23, 25, 26, 40–50]. For example, when the aphid symbiont *H. defensa* harbors a lysogenic lamdoid bacteriophage, the *Acyrthosiphon pisum* secondary endosymbiont (APSE; [10]), it provides varying degrees of protection from some species of parasitoid wasps, but not others [10, 19, 24]. *Hamilitonella* with the APSE bacteriophage protects the pea aphid against parasitism by *Aphelinus ervi* and *Aphelinus abdominalis* wasps, but it doesn’t appear to affect the survival of another pea aphid parasitoid wasp *Praon pequodorum*. This suggests that the protective toxin produced by the APSE bacteriophage does not provide general protection against all of *A. pisum’*s parasitoids [22]. Furthermore, several recent studies have demonstrated that *Hamiltonella*’s protection against parasitoids is not limited only to its mututalism with *A. pisum.* A second species of aphid, the cowpea aphid, *Aphis craccivora* is also protected from parasitism by two parasitoid wasps, *Binodoxys communis* and *Binodoxys koreanus* when it harbors *Hamiltonella* but were just as likely to be parasitized by *Lysiphelbus orientalis* and *Aphidius colemani* as symbiont free cowpea aphids. This suggests a genus-level specificity of protection in this system [44]. Interestingly, in a third aphid species, the grain aphid *Sitobion avenae,* individuals harboring *Hamiltonella* are equally susceptible to parasitism by *A. ervi* and *Ephedrus plagiator* as those without the symbiont, however, female wasps from both parasitoid species prefer to lay their eggs in symbiont-free aphids when given the choice. In this case, while *Hamiltonella* may not increase resistance against parasitism, it is still protecting its aphid host by making it less attractive to multiple parasitoid species [20]. Taken together, these studies suggest that even a single symbiont, in this case *Hamiltonella*, does not provide aphids with general protection against all parasitoid wasps. Our current study is the second to find negative fitness effects of aphid symbionts on an aphidophagous lady beetle. We have now documented this in two beetles in the family Coccinellidae. Future studies should be done to determine how general these fitness effects are across other aphidophagous predators such as lacewings or predatory midges.

In conclusion, our current study finds that two aphid secondary symbionts can significantly impact the survival of the predatory lady beetle *Ha. axyridis* during the larval and pupal development periods. They can also increase the length of development time and influence adult weight. All of these effects can have significant fitness consequences for aphid predators, which may in turn significantly affect the fitness of their aphid prey. During the summer months, parthenogenetically reproducing aphids live in clusters of highly related clone-mates with low rates of dispersal. Under these conditions we could expect lower predator survival to result in a lower overall risk of predation for a population of genetically identical aphids [28]. And while the fitness effects are similar to those found in a previous study in another species of lady beetle, there does appear to be some variation in how each symbiont affects each predator species. Finally, these findings demonstrate the far-reaching effects of symbionts beyond just host fitness and survival, and suggest that symbionts have important ecological impacts along multiple food chains and across food webs.

## Additional file

**Additional file 1: Data S1.** Raw experimental data.

## Abbreviations

5AU: aphids from the 5A clonal line that harbor the *Regiella insecticola* facultative symbiont
5AT: aphids from the 5A clonal line that harbor the *Hamiltonella defensa* facultative symbiont
5AO: aphids from the 5A clonal line that do not harbor any facultative symbionts
5AR: aphids from the 5A clonal line that harbor the *Serratia symbiotica* facultative symbiont
